# 1247. Going Viral with Antimicrobial Stewardship

**DOI:** 10.1093/ofid/ofad500.1087

**Published:** 2023-11-27

**Authors:** Abi Jenkins, Jubeyr Ahmed, Andrew Bosworth, I Michael Kidd, Husam Osman, Sowsan F Atabani

**Affiliations:** University Hospitals Birmingham, Birmingham, England, United Kingdom; Microbiology Services, UKHSA Birmingham Laboratory and University Hospitals Birmingham, Birmingham, England, United Kingdom; Microbiology Services, UKHSA Birmingham Laboratory and University Hospitals Birmingham, Birmingham, England, United Kingdom; Microbiology Services, UKHSA Birmingham Laboratory and University Hospitals Birmingham, Birmingham, England, United Kingdom; Microbiology Services, UKHSA Birmingham Laboratory and University Hospitals Birmingham, Birmingham, England, United Kingdom; Public Health Laboratory Birmingham, Birmingham, England, United Kingdom

## Abstract

**Background:**

Historically, antimicrobial stewardship (AMS) has considered the judicious use of antibiotics. AMS is widely adopted across Europe and the US; recently antifungal AMS is gaining momentum but antiviral AMS has been little described. Here we report findings following introduction of AMS virology reviews at University Hospitals Birmingham (UHB); a novel concept and an opportunity to broaden the beneficial aspects of AMS to virology.

**Methods:**

In June 2022, a UK supply issue with aciclovir (ACV) injection was announced. In order to review and preserve ACV injection for those in greatest need, UHB virologists implemented a specialist review for all patients prescribed > 48 hours of treatment. Additionally, we collected data on the advice offered, whether it was accepted, and time required completing the review.

**Results:**

ACV usage had no seasonal pattern. Over the shortage period of 10 weeks, AMS virology rounds were conducted twice weekly either face-to-face or remotely via the Electronic Patient Management System. During these 10 weeks, IV ACV administered to patients for more than 48 hours of treatment was reduced by 51%. Depending on the patient complexity, ward rounds lasted between 60 and 120 minutes (n=24) with advice given to cease treatment (n=8), IV-to-oral switch (n=5), to continue IV ACV (n=8) and/or to undertake further sampling and diagnostic testing (n=6). Advice offered by the clinical virologists to the host clinicians was accepted in all cases.

**Conclusion:**

Antiviral AMS rounds halved IV ACV consumption, compared to pre or post intervention levels, with more than half of patients advised to stop or switch to oral therapy. Diagnostics and sampling guidance was offered in one quarter of reviews, whilst the remaining interventions were more stewardship focused. In all cases stewardship advice was readily accepted by clinical teams. Antiviral AMS rounds provide an opportunity to optimise sampling, diagnosis and improve patient management. Introduction of regular antiviral AMS rounds at UHB are at advanced planning.

**Disclosures:**

**All Authors**: No reported disclosures

